# Aging Negatively Affects Estrogens-Mediated Effects on Nitric Oxide Bioavailability by Shifting ERα/ERβ Balance in Female Mice

**DOI:** 10.1371/journal.pone.0025335

**Published:** 2011-09-22

**Authors:** Laura Novensà, Susana Novella, Pascual Medina, Gloria Segarra, Nadia Castillo, Magda Heras, Carlos Hermenegildo, Ana Paula Dantas

**Affiliations:** 1 Institut d'Investigacions Biomèdiques August Pi i Sunyer (IDIBAPS), Barcelona, Spain; 2 Institut Clinic de Tòrax, Hospital Clinic Barcelona, Spain; 3 Department of Physiology, University of Valencia, Valencia, Spain; 4 Research Foundation, Hospital Clínico Universitario, Valencia, Spain; 5 Instituto de Investigación Sanitaria INCLIVA, Hospital Clínico Universitario, Valencia, Spain; University of Valencia, Spain

## Abstract

**Aims:**

Aging is among the major causes for the lack of cardiovascular protection by estrogen (E2) during postmenopause. Our study aims to determine the mechanisms whereby aging changes E2 effects on nitric oxide (NO) production in a mouse model of accelerated senescence (SAM).

**Methods and Results:**

Although we found no differences on NO production in females SAM prone (SAMP, aged) compared to SAM resistant (SAMR, young), by either DAF-2 fluorescence or plasmatic nitrite/nitrate (NO2/NO3), in both cases, E2 treatment increased NO production in SAMR but had no effect in SAMP. Those results are in agreement with changes of eNOS protein and gene expression. E2 up-regulated eNOS expression in SAMR but not in SAMP. E2 is also known to increase NO by decreasing its catabolism by superoxide anion (O_2_^-^). Interestingly, E2 treatment decreased O_2_^−^ production in young females, while increased O_2_^−^ in aged ones. Furthermore, we observed that aging changed expression ratio of estrogen receptors (ERβ/ERα) and levels of DNA methylation. Increased ratio ERβ/ERα in aged females is associated to a lack of estrogen modulation of NO production and with a reversal in its antioxidant effect to a pro-oxidant profile.

**Conclusions:**

Together, our data suggest that aging has detrimental effects on E2-mediated benefits on NO bioavailability, partially by affecting the ability of E2 to induce up regulation of eNOS and decrease of O_2_^−^. These modifications may be associated to aging-mediated modifications on global DNA methylation status, but not to a specific methylation at 5′flanking region of ERα gene.

## Introduction

The surprising controversies on the risk-benefits of estrogen replacement therapy (ERT) may, in part, be explained by age disparity among studies. While the cardiovascular effects of estrogen in experimental studies have been determined mostly in young ovariectomized females, clinical trials have been carried out on aged women, who were on average, 10 years past the onset of menopause[1], [2]. Aging, *per se,* is known to cause a series of alterations in the endogenous mechanisms that control cardiovascular function leading to subsequently increased risk of cardiovascular disease[3], [4], [5]. Detailed examination of the data from the Woman's Health Initiative (WHI) indicates that early initiation of estrogen replacement produces more favorable results than the late average time of initiation employed in the WHI studies overall [6], [7], [8]. These observations together with other observational studies have led scientists to create the so-called “Timing Hypothesis”. This theory states that estrogen-mediated benefits to prevent cardiovascular disease may occur only when treatment is initiated before the detrimental effects of aging on cardiovascular disease are established in the vasculature[9].

Since this is a relatively recent theory, little information is available on whether and how vascular effects of estrogen are modified with aging in females. Few studies have shown that aging in female rodents is associated with significant reduction of estrogen-mediated cardiovascular effects. The lack of estrogen responses in those animals was not related to age-associated changes in the plasma levels of estrogen or activity of estrogen receptors, but rather by possible age-related changes in estrogen-mediated signaling pathways in the vasculature[10], [11], [12]. Moreover, recent clinical studies have revealed that different risk factors for cardiovascular disease in postmenopausal women were lower among women 50 to 59 years old at enrollment for ERT[13], [14]. These studies demonstrate that estrogen has complex biologic effects and may influence the risk of cardiovascular events and other outcomes through multiple pathways. Nevertheless, the field lacks detailed research on the long-term effects by estrogen and how it modulates cardiovascular function during aging. It remains unclear to what extent the protective effects of estrogen replacement well described in young females can be extrapolated to older ones. Cardiovascular disease is the number one killer of women in the Western world. Thus, it is imperative we understand the mechanisms underlying why the vast number of experimental studies indicate estrogen replacement is cardioprotective while no beneficial effect was observed in clinical trials such as the WHI. To address this important issue, this study aims to determine how aging can affects nitric oxide production by estrogen in female vasculature.

## Methods

Female senescence-accelerated resistant (SAMR, young) and senescence-accelerated prone (SAMP, aged) mice obtained from the breeding stock at Parc Cientific de Barcelona were housed at Animal Facility of University of Barcelona according to institutional guidelines (constant room temperature — 22°C, 12 h light/dark cycle, 60% humidity, standard mice chow and water *ad libitum)*. All protocols were approved by the Institutional Ethics Committee at the University of Barcelona (Comitè Ètic d'Experimentació Animal – CEEA protocol: 625/08), conformed to the National Institute of Health Guide for the Care and Use of Laboratory Animals. SAM model was chosen based on previous studies from our group establishing SAMP-OVX as a good model to concomitantly study the effects of aging and menopause in middle-aged female mice [15]. Both SAMR (n = 40) and SAMP (n = 45) were randomly separated at 7 months of age into two groups: 1) ovariectomized (OVX); and 2) ovariectomized chronically-treated with estrogen (OVX+E2). This age was chosen based on preliminary studies to determine the beginning of vascular senescence in SAMP, but not in SAMR[15], [16]. Ovariectomy was performed under controlled inhalant anesthesia with isoflurane (4% induction and 1.5–2% maintenance). After ovariectomy, mice were treated either with vehicle or 17β-estradiol in mineral oil solution (5 µg/kg) by subcutaneous injection every three days in order to simulate the estral cycle[17]. The efficacy of ovariectomy and estrogen treatment was determined by uterine weight. Four weeks following ovaricetomy, all mice were euthanized by anesthesia with isoflurane and aorta was harvested from the aortic arch to the common iliac artery. In order to establish a correlation among results, in a set of animals half of the aorta was gradually frozen in Tissue-Tek® OCT compound (for protocols in aortic section), and the other half was snap frozen into liquid nitrogen (for mRNA and DNA analysis).

### Nitric Oxide Measurements

Because of its short half-life, NO production was determined by both intracellular nitrosilation of NO-sensitive fluorochrome DAF-2 in aortic sections and by measuring the concentration of NO metabolites nitrite (NO_2_) and nitrate (NO_3_) in plasma. For DAF-2, aortic sections (4 µm) were incubated with a permeable form DAF-2 DA (5 µM, 30 min, 37°C) and kept in the dark at 37°C with a warming stage on an inverted confocal microscope (Axiovert 2000, Carl Zeiss Inc). DAF-2 fluorescence was assessed basally at excitation/emission wavelength of 495/515 and after challenging with carbachol (10 nM to 100 µM) to increase NO production, with readings taken 5 min after exposure to the agonist. Images were recorded by Axiovision 4.6 software (Zeiss Imaging) and increase of green fluorescence intensity by the distinct groups of animals was measured with Mac Biophotonic ImageJ Software. Data were expressed as fold increase following carbachol stimulation. The levels of NO metabolites (NO_2_/NO_3_) were determined in the plasma by a commercial colorimetric assay kit (Cayman Chemical Company) following the supplier's instructions.

### NAD(P)H oxidase Activity

Lucigenin-enhanced chemiluminescence was used to determine superoxide production after adding excess NADPH (100 µM), the substrate for NAD(P)H oxidase, as described [18]. This method is specifically designed to measure NAD(P)H oxidase capacity. Freshly isolated aortas were placed into distinct centrifuge tubes containing Krebs-Henseleit solution with lucigenin (5 µM) and kept at 37°C. Increase of luminescent signal following addition of 100 µM NADPH was determined in a luminometer (Berthold AutoLumat Plus) every 2 seconds for a 3-minute period. Background counts (determined in tissue-free preparations) were subtracted from images, and values were normalized to protein content (BCA assay).

### Immunofluorescence

Protein expression and localization of eNOS and ERs (ERα and ERβ) were determined in aortic sections from SAMR and SAMP mice by immunofluorescence as previously described[15]. Aortic sections (4 µm) were thaw-mounted onto polylysine covered slides, fixed in acetone (15 min) and blocked for 30 min with horse serum. Sections were incubated overnight at 4°C with primary antibodies: 1∶200 anti-eNOS (Abcam - ab5589), 1∶100 anti-ERα (Abcam - ab37438) or 1∶50 anti-ERβ (Abcam - ab3576). Following washes, sections were co-stained with 10 µM phalloidin (Sigma) and secondary antibodies 1∶500 Alexa Fluor 488 conjugated goat anti-rabbit (Invitrogen) for eNOS or 1∶500 Alexa Fluor 647 conjugated donkey anti-rabbit (Invitrogen) for ERs. Coverslips were mounted on slides using ProLong Gold antifade reagent with DAPI (Invitrogen), and sections were visualized through a confocal microscope (Axiovert 2000, Carl Zeiss Inc) with a 40X objective lens (Zeiss). For each image, light was passed through a different excitation filter: 1) 350 nm (for DAPI); 2) 490 nm (for Alexa 488); 3) 590 nm (for phalloidin) and 4) 650 nm (for Alexa 647). Each aorta was recorded in 3 different regions and results were expressed as an average of fluorescence elicited using Mac Biophotonic ImageJ Software.

### Western blot analysis

Equal amount of protein from each sample (50 µg) were resolved by SDS-PAGE on 4-12% gels and electroblotted onto nitrocellulose. After 1 h blocking with 5% milk in Phosphate-buffered saline with 0.1% (v/v) Tween 20 (PBST), membranes were incubated for 1 h in PBST containing 5% milk and the specified primary antibody: monoclonal anti eNOS, 1∶1000 (BD Transduction Laboratories). After 4 washes with PBST, membranes were incubated for 1 h with a horseradish peroxidase-labeled goat anti-mouse at a 1∶2000 dilution in PBST containing 1% milk. After 4 additional washes in, the membranes were incubated with a chemiluminescent reagent according to the manufacturer's protocols (SuperSignal west pico, Pierce Chemical Co), and chemoluminescent signal was visualized by LAS3000 imaging system (Fujifilm). Densitometric analyses of Western blots were performed using a Mac Biophotonic ImageJ Software. All membranes were reblotted using a monoclonal antibody anti GAPDH (1∶2500; Santa Cruz Biotechnology) as a loading control. Data were normalized to corresponding values of GAPDH densitometry.

### Quantitative Real-Time PCR (qRT-PCR)

Total RNA was isolated and reverse transcribed as previously described [19]. mRNAs encoding eNOS, the subunities of NAD(P)H-oxidase (NOX1, p47^phox^, p22^phox^) and ER (ERα, ERβ) were quantified by qRT-PCR based on SYBR® Green fluorescence, using the 18S ribosomal subunit of RNA (Inventoried: Hs99999901 s1, Applied Biosystems) as internal control. The specific primer sequences for mice are described on Table 1. Real-time PCR reactions were set following the manufacture's conditions (Applied Biosystems).

**Table 1:** Primers designed for qPCR experiments.

Gene	Sequence(5′→3′)
eNOS (NM_008713.4)	*F: TGTCACTATGGCAACCAGCGT* *R: GCGCAATGTGAGTCCGAAAA*
NOX1 (NM_172203.1)	*F: CCTTCCATAAGCTGGTGGCAT* *R: GCCATGGATCCCTAAGCAGAT*
p22^phox^ (NM_007806.3)	*F: GGCCATTGCCAGTGTGATCTA* *R: TGCTTGATGGTGCCTCCAA*
p47^phox^ (NM_010876.3)	*F: AGGAGATGTTCCCCATTGAGG* *R: CAGTCCCATGAGGCTGTTGAA*
ERα (NM_007956.4)	*F: TGCCTGGCTGGAGATTCTG* *R: CTTCCCCGGGTGTTCCAT*
ERβ (NM_207707.1)	*F: CAGGCTGAGCGACAACCA* *R: CTCTAAATGCAGACACGTACTTTCCT*
ERα 5′ flanking region (MSP)(AJ276597.1)	*F: GTTAAGGGGAATTATGCGTGC* *R: TTACAAACTAACTCCCCGCG*
ERα 5′ flanking region (No MSP)(AJ276597.1)	*F: GTGTTAAGGGGAATTATGTGTGT* *R: TTACAAACTAACTCCCCACACA*

### Analysis of Global Methylation

Global DNA methylation status was determined by non-isotopic cytosine extension assay, as previously described[20]. Briefly, equal amounts of genomic DNA (500 ng), isolated from total aorta underwent no digestion (Mock) or digestion with 10 U of methylation-sensitive restriction enzyme HpaII or its isoschizomer MspI (New England Biolabs) for 16–18 hours in separate tubes. Digested DNA was subjected to single-nucleotide extension reaction in a 20 µl reaction containing Taq polymerase (Promega) and 5 µl of 0.4 mM biotin-14-dCTP (Invitrogen) for 1 h at 56°C. 2 µl in triplicate of each sample were applied on a nylon membrane for DNA incorporation. After washing with 0.4 N NaOH and cross-link at U.V. light, biotin incorporated to the membranes was visualized with HRP-streptavidin reaction (Pierce), according to manufacturer. Values were corrected for non specific incorporation by measuring the incorporation of biotin-dCTP into undigested genomic DNA (Mock samples). The difference in the mean incorporation of biotin-dCTP following MspI and HpaII was computed as the global DNA methylation level and expressed as % of DNA methylation.

### Methylation Sensitive qRT-PCR

The methylation status of 5′ flanking region of the gene encoding ERα was determined by methylation specific qPCR, as described [21]. Following mock digestion or enzymatic digestion with HpaII or MspI, resulting DNA was amplified using qPCR with Power SYBR® Green master mix as described by the manufacturer (Applied Biosystems). PCR primers were designed using Methyl Primer Software (Applied Biosystems) to amplify the CpG island that spans the 5′ flanking region of ERα gene (MSP) or to amplify a region that is devoid of any of the restriction sites of the enzymes used in the design of the experiment (No MSP), as internal control (Table 1). Methylation at CpG sites prevents HpaII, but not MspI, digestion and allows the amplification of the fragment, resulting in a low cycle threshold (Ct) value. In contrast, if the CpG island is not methylated, HpaII cleaves DNA and prevent amplification of the fragment, resulting in higher Ct values. Each sample was analyzed in duplicate.

### Data Analysis

Data are expressed as means ± SEM obtained from 6–8 individual aortic samples from each animal group. Differences between mouse strains (i.e SAMR vs. SAMP) and by experimental treatment groups (i.e., OVX vs. OVX+E2) were analyzed by two-way ANOVA, followed by Bonferroni's post-test to compare replicate means. Association between increase of ERβ/ERα ratio and changes on NO production and NOX1 expression in SAMR and SAMP animals was calculated by Pearson's Correlation Coefficient (r). Statistical significance was accepted at p<0.05. Statistical analysis and Pearson's Coefficient calculation were carried out using Graph Pad Prism v5 software (GraphPad Software Inc., San Diego, CA, USA).

## Results

**Figure 1:**
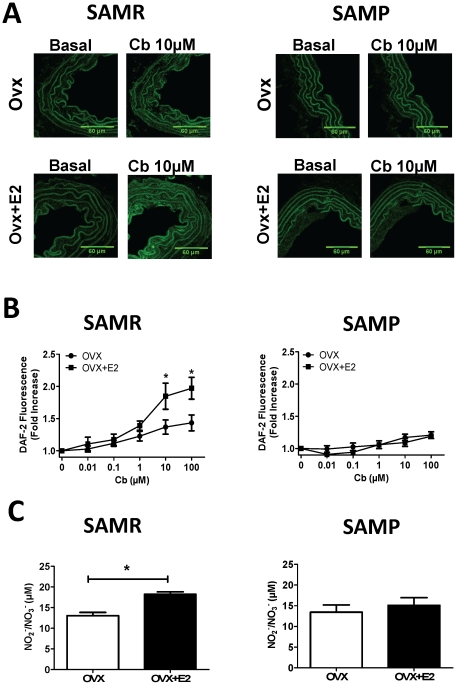
NO production in young (SAMR) and aged (SAMP) female mice. (**A**) representative images of DAF2-DA fluorescence in mice aorta at basal level and after challenging with carbachol (Cb) 10 µM; (**B**) dose response, shown as relative green fluorescence intensity (fold increase) following carbachol stimulation; (**C**) nitrite/nitrate (NO_2_/NO_3_) concentration (µM) in plasma from SAMR and SAMP mice. OVX, ovariectomized; OVX+E2, ovariectomized treated with estrogen. Data are plotted as the mean ± S.E.M derived from 6–8 independent experiments. * p<0.05.

**Figure 2:**
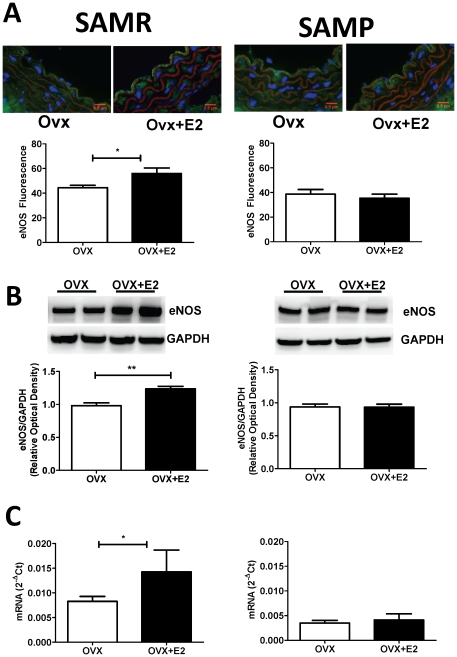
eNOS expression in thoracic aorta from SAMR and SAMP mice. (**A**) top: representative immunofluorescent merged images. Staining shows nucleus (blue, DAPI), actin fibers (red, phalloidin), eNOS (green). Bar graphs (bottom) show the results of densitometric analyses from pooled data. (**B**) Immunoblots analysis (top) in single aortas probed with antibodies against eNOS or GAPDH, as indicated. The lower graph shows the results of densitometric analyses from pooled data, plotted as optical densitometry relative to the signal obtained in by GAPDH. (**C**) eNOS mRNA expression in mice aorta normalized to the expression of ribosomal RNA subunit 18S, which was used as an endogenous reference gene. OVX, ovariectomized; OVX+E2, ovariectomized treated with estrogen. Each data represents the mean ± SEM derived from 6–8 independent experiments. * p<0.05.

**Figure 3:**
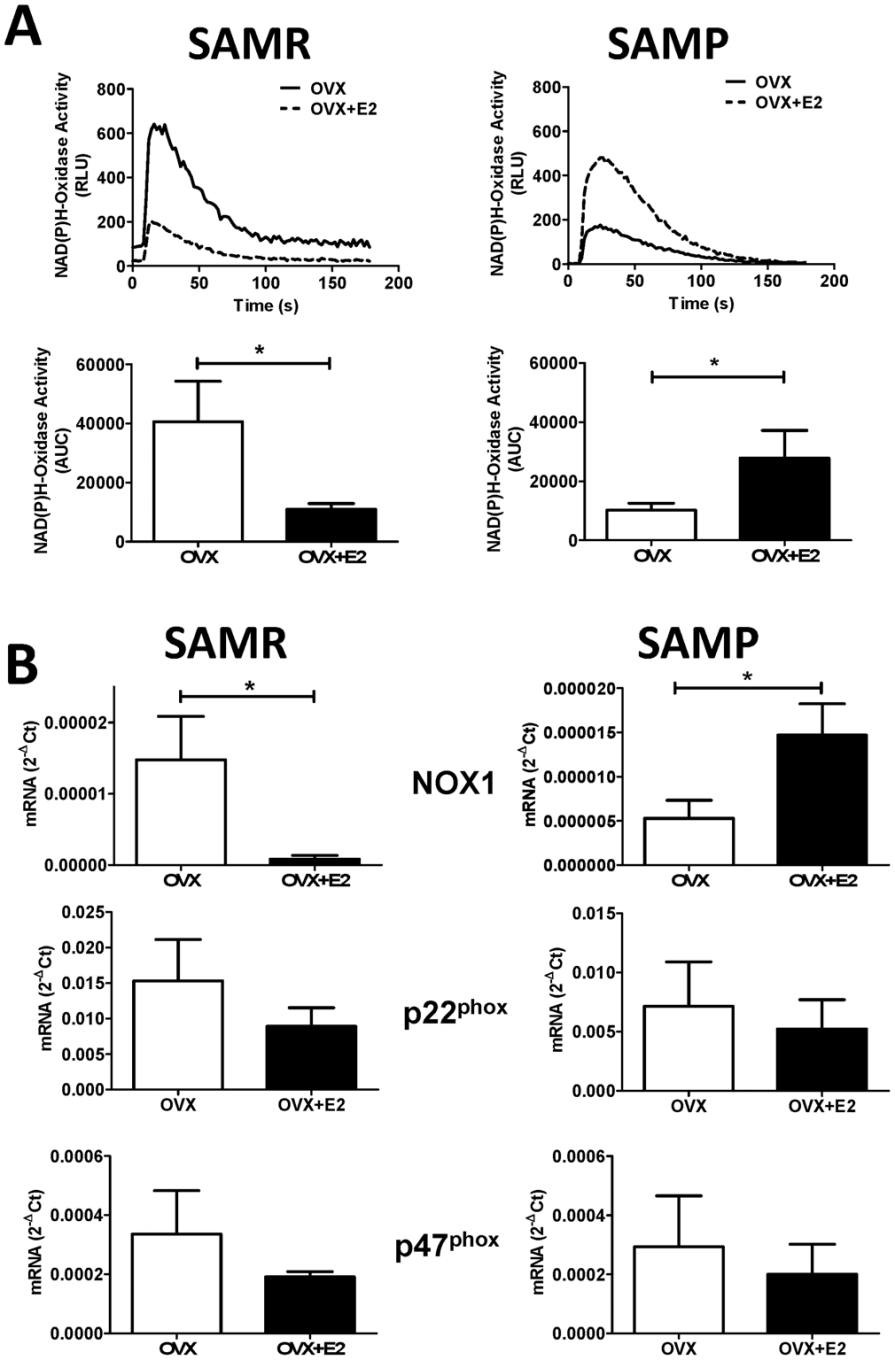
NAD(P)H-dependent O_2_^-^ production in aorta from young (SAMR) and aged (SAMP) females. (A) On top: Representative chemiluminescence tracing of NADPH-dependent O_2_^−^production in SAMR and SAMP mice. On the bottom bar graphs represent mean ± SEM of the areas under the curve obtained from 6–8 individual experiments. (B) mRNA expression of the NAD(P)H oxidase subunits in mice aorta normalized to the expression of ribosomal RNA subunit 18S, used as an endogenous control. OVX, ovariectomized; OVX+E2, ovariectomized treated with estrogen. Each data represents the mean ± SEM derived from 6–8 independent experiments. * p<0.05.

We observed important differences on estrogen-mediated effects on NO production between young (SAMR) and aged (SAMP) mice. NO production was not significantly modified by aging in the absence of estrogen in both methods of DAF-2 (Max Fold Increase: SAMR 1.4±0.07 vs. SAMP 1.2±0.04; p = ns) and NO_2_/NO_3_ (µM: SAMR 13.1±0.8 vs. SAMP 13.5±1.7 p = ns). However, treatment with estrogen was effective to increase NO production only in younger females. A similar pattern of estrogen-induced increase of NO was observed in aorta (33.7%) and in plasma (28.3%) of SAMR OVX, whereas no effect was observed in aged (SAMP) OVX (Figure 1). It is known that the major mechanism involved on estrogen-induced increase in NO availability includes transcriptional modulation of eNOS expression. Therefore, aging-associated decrease of estrogen effect may be, in part, associated to a diminished action on the modulation of nitric oxide synthase (eNOS) expression.Both immunofluoresce (Figure 2A) and Western blot (Figure 2B) analysis reveal that estrogen treatment is able to increase eNOS protein expression in aortas of SAMR, but not of SAMP OVX. Those results are in agreement with results from mRNA expression (Figure 2C) showing that estrogen treatment is effective to modulate eNOS gene expression only in SAMR females. In addition, the decreased estrogen-mediated modulation of NO in aged females could be explained by a change on NO catabolism. The biological activity of NO is modulated by changes in reactive oxygen species (ROS), such as superoxide anion (O_2_^−^) [22]. Because estrogen has been described to increase NO by an antioxidant mechanism that involves down-regulation of NAD(P)H oxidase, we have also explored the potential role of aging on estrogen-induced modulation of NAD(P)H oxidase activity and expression. In preliminary studies, lucigenin-luminescence was determined in the presence of apocynin (10 µM) and by using xanthine(100 mM) as a substrate. In both cases the superoxide-induced increase in luminescence was blunted, confirming that NADPH-oxidase is the major source for superoxide generation in aorta from SAM mice (data not shown). Curiously, although we have observed in previous studies that aging *per se* markedly increases oxidative stress in our mice model, we found in these studies that estrogen plays a negative role on O_2_^−^ modulation. As can be seen on Figure 3A, young OVX exhibits a much higher NAD(P)H oxidase-associated superoxide generation than do aged females (AUC: SAMR 40596±13680 vs. SAMP 10153±2357, p<0.05). Furthermore, and even more intriguing is the finding that estrogen treatment acts as an antioxidant in young females while has pro-oxidant effects in old females (Figure 3A). These responses can be associated to a reversal of estrogen-mediated action on the expression of NAD(P)H oxidase subunities, as we have observed that estrogen has opposite effects on the modulation of NOX1 subunit in SAMR OVX in comparison to SAMP OVX (Figure 3B). We observed that aged OVX expresses much lower levels of NOX1 than do young ones (mRNA 2^−ΔCt^: SAMR 1.5×10^−5^±6.1×10^−6^ vs. SAMP 5.3×10^−6^±2.1×10^−6^; p<0.05), even though no aging-associated differences were observed on the expression of p22^phox^ and p47^phox^subunits. Estrogen treatment of OVX mice markedly decreased NOX1 expression in SAMR (8.0×10^−7^±5.5×10^−7^; p<0.05), but increased NOX1 expression in SAMP OVX to the levels observed in untreated SAMR OVX (1.4×10^−5^±3.6×10^−6^; p<0.05). Although a trend of decrease on the expression of p22^phox^ and p47^phox^ was observed in both estrogen-treated groups, no statistical significances were found (Figure 3B), suggesting that NOX1 is the major responsible for the responses on superoxide generation observed herein.

**Figure 4:**
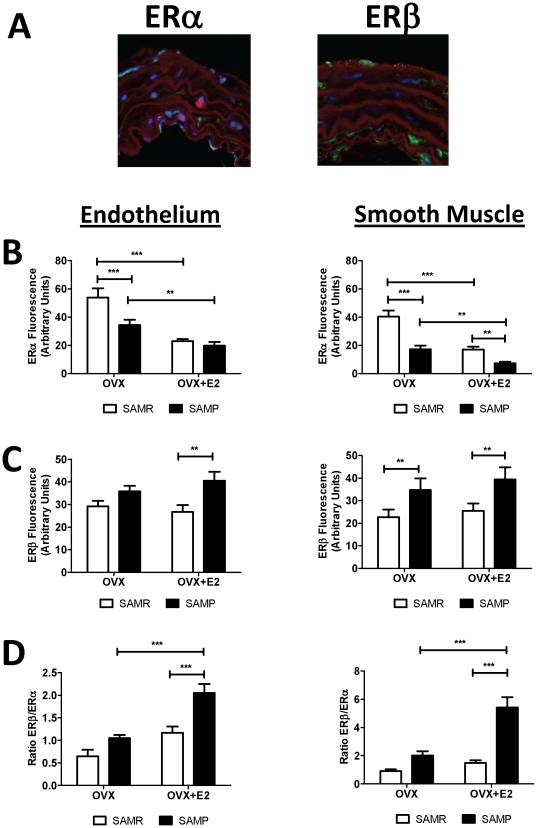
Estrogen receptor (ERα and ERβ) expression in thoracic aorta from SAMR and SAMP mice. (**A**) representative immunofluorescent merged images shows nucleus (blue, DAPI), actin fibers (red, phalloidin), ER (green: not merged with nucleus or purple: merged with nucleus). Bar graphs show the results of densitometric analyses from pooled data for ERα (**B**) and ERβ (**C**) expression in both vascular endothelium and smooth muscle. (**D**) Ratio of ERβ/ERα expression in both endothelium and vascular smooth muscle. OVX, ovariectomized; OVX+E2, ovariectomized treated with estrogen. Each data represents the mean ± SEM derived from 6–8 independent experiments. ** p<0.01 and *** p<0.001.

**Figure 5:**
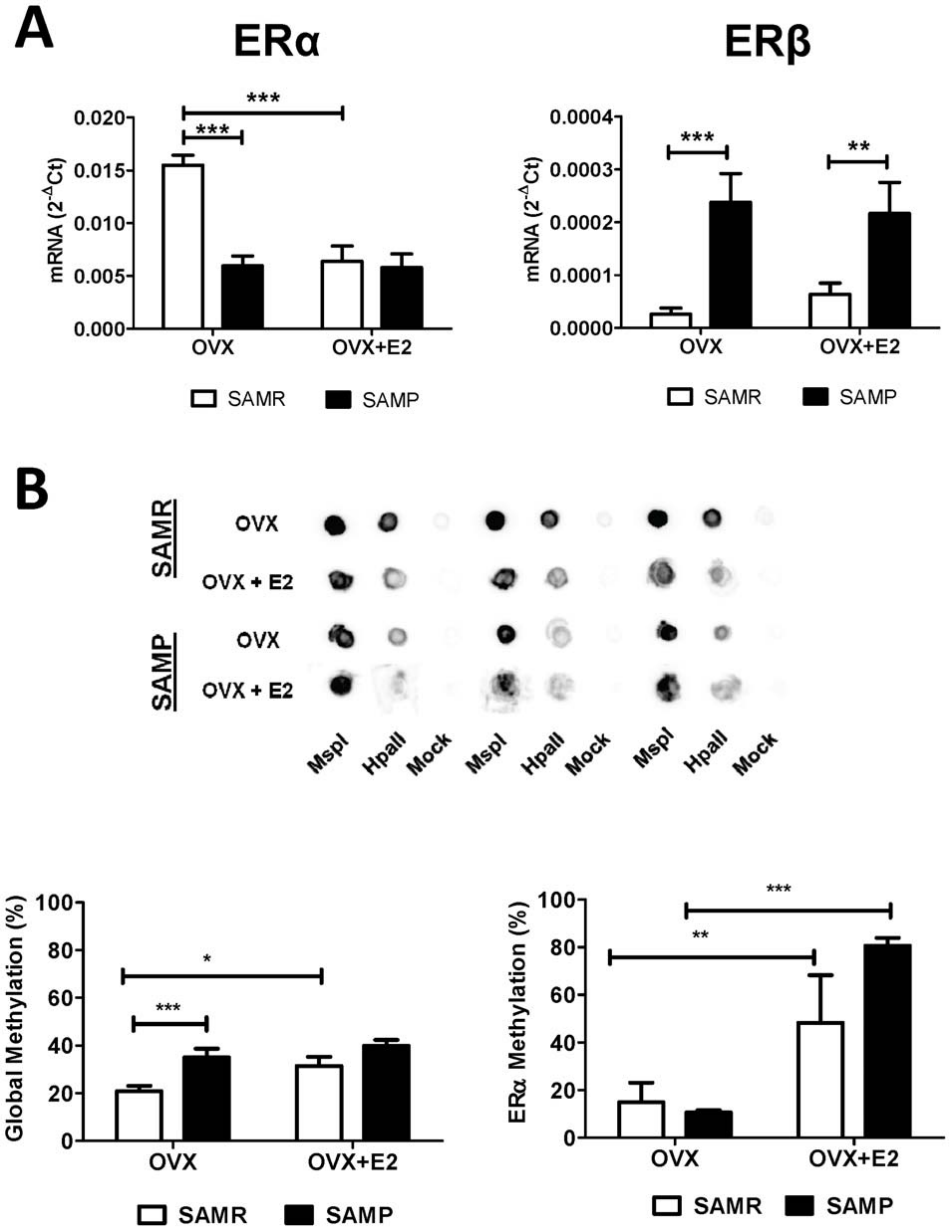
Aging-associated change on ER expression and DNA methylation. (**A**) mRNA expression for ERα and ERβ in mice aorta normalized to the 18S expression. (**B**) Image on top shows a representative experiment of biotin-dCTP incorporation to DNA following no digestion (Mock) and digestion with MspI (no methylation sensitive) or HpaII (methylation sensitive).DNA was spotted in a nylon membrane and visualized with streptavidin-HRP method (data shown in triplicate). Bar graphs show (left) the results of densitometric analyses from pooled data for global DNA methylation and (right) percentage of specific DNA methylation at 5′ flanking region of the gene encoding ERα. OVX, ovariectomized; OVX+E2, ovariectomized treated with estrogen. Each data represents the mean ± SEM derived from 6–8 independent experiments. * p<0.05; ** p<0.01 and *** p<0.001.

Most of the vascular protective effects induced by estrogens are mediated by a direct genomic action through interaction with its receptors: ERα and ERβ. We next explored a potential role of aging-associated change on the expression of ERs and its potential correlation with the altered responses on NO/O_2_^−^ production. On Figure 4 we show the results of immunofluorescence analysis that determined the expression of ERα and ERβ on the endothelium and smooth muscle cells from SAMR and SAMP aortas. In these studies we observed that ERα is the major receptor expressed in mice aorta (Figures 4B and 4C), and it is present at higher level at the endothelium in comparison to the smooth muscle layer (Figure 4B). Aging *per se* notably alters ERs expression in both vascular endothelium and smooth muscle in aortas from OVX mice, as expressed by increased ratio of ERβ/ERα (Figure 4D). Although no statistical significance was found on ERβ/ERα ratio in OVX mice, SAMP OVX shows a marked decrease of ERα and a slight increase of ERβ expression in both endothelium and smooth muscle. Estrogen treatment decreases ERα expression in both SAMR and SAMP females, but has no effect on ERβ. The magnitude of reduction on ERα after treatment with estrogen in SAMR is more than that in SAMP, and is reflected by a significant increase of ERβ/ERα in aged, but not in young females (Figure 4C). Besides, the changes on ERβ/ERα ratio observed on aortic smooth muscle are exceedingly higher than that found on endothelium (Figure 4C). Comparable results on mRNA expression for ERs were found on by qRT-PCR (Figure 5A). These studies confirm that ERα is expressed at much higher level than ERβ in mice aorta, and show that both aging and estrogen down regulates ERα by negatively modulating its transcription. Interestingly, although ERβ mRNA expression was also augmented in aged groups, the magnitude of increase on mRNA was much higher (Figure 5A) than that observed for protein expression (Figure 4C). Furthermore, Pierson's Correlation analysis reveals no linear relationship between changes in ERβ/ERα ratio and nitric oxide production (Table 2). In contrast, a significant positive correlation is seen between ERβ/ERα ratio and NOX1 expression in SAMP females (Table 2). These data establishes that the marked increase of ERβ/ERα in SAMP OVX+E2 is responsible for the increased oxidative stress observed.

**Table 2:** Pierson Correlation Coefficients between ERβ/ERα ration and changes in NO or O_2_^−^ systems.

	SAMR			SAMP		
	Pierson r	P value	Significance	Pierson r	P value	Significance
NO (DAF2)	0.434	0.062	ns	0.383	0.128	ns
eNOS IF	0.513	0.087	ns	−0.221	0.410	ns
eNOS mRNA	0.473	0.102	ns	−0.151	0.575	ns
Endo (ERβ/ERα)vs NOX1 mRNA	0.409	0.239	ns	0.938	<0.001	***
SMC (ERβ/ERα)vs NOX1 mRNA	0.407	0.243	ns	0.951	<0.001	***

Alterations in the heritable patterns of DNA methylation have been associated to the decrease of gene expression and protein activity [23]. To test this hypothesis, we evaluated alteration in global DNA methylation density within CpG islands and in specific CpG islands on 5′ flanking region of ERα. In these studies, we found that both aging and estrogen treatment affects the levels of global methylation in female mice. An increase on percentage of global DNA methylation was observed in SAMP OVX in comparison to SAMR OVX. Estrogen treatment similarly enhanced the levels of DNA methylation in SAMR OVX, although no further increase on DNA methylation was observed in SAMP OVX (Figure 5B). Studies by methylation-sensitive qPCR showed increased percentage on DNA methylation at 5′ flanking region of the gene encoding ERα in OVX treated mice from both SAMR and SAMP groups (Figure 5B). No differences on ERα DNA methylation were observed when SAMR vs. SAMP were compared.

## Discussion

In this study we investigated the hypothesis that aging has a detrimental effect on the modulatory role of estrogen to regulate nitric oxide bioavailability in female aorta. To date, few studies have addressed to the effects of aging on estrogen responses using rodent models at advanced age. Aging has shown to modify estrogen-mediated benefits on vascular function in middle aged female rats with risk for cardiovascular disease[24], [25]. On the other hand, in female rodents that normally do not exhibit cardiovascular complications, aging-associated changes in vascular function and estrogen effects are evident only when animals are senile[11], [12], [26]. The fact that those studies used animal models normally display increased risk for cardiovascular disease at early stage or used senile females, they may not represent the modifications that are associated with aging and menopause in women.

In previous studies we have established ovariectomized senescent accelerated prone (SAMP OVX) mice as a fine model to study progressive cardiovascular aging in females concomitantly with loss of ovarian function. At age of six months (middle age) SAMP OVX exhibits a significant decrease of endothelium-dependent and NO-mediated relaxation [15]. The fact that those effects were not as evident on senescent accelerated resistant mice (SAMR OVX), suggests an overlap between aging and hormonal status to control endothelial function, that closely mimics vascular dysfunction observed in menopausal women[27], [28]. In view of the fact that SAMP OVX at 6 months is characterized as middle aged female that have not reached a high level of vascular damage, we have found that estrogen treatment is still effective to improve endothelium-dependent relaxation[16]. In these studies we sought to determine if the progression of age affects estrogen-mediated responses on the modulation of nitric oxide. For that, we used our model of menopause (SAMP OVX) at 7–8 months of age following immediate replacement with 17β-estradiol or placebo. We carefully considered the use of an intact control but our preliminary studies showed that, like other rodents, SAM mice do not reach reproductive senescence until later in life [29]. At 6–8 months SAMR and SAMP exhibit similar hormonal status with sustained estrus and slowly declining levels of estrogens [15], [29], and therefore the addition of these multiple groups would complicate analysis and make it more difficult to discern the effect of aging on E2 effects, which was the main question of interest.

A correlation of aging and decrease of endothelium-dependent and nitric oxide-mediated has been extensively described in both man and women[14], [30], however limited information is available on the effects of aging on estrogen-mediated action. We found that aging (at this point) significantly affects estrogen-mediated mechanisms that modulate nitric oxide bioavailability. While young females SAMR OVX shows estrogen benefits by up-regulation of eNOS expression and decrease of oxidative stress, an interesting reversal in the estrogen effects was observed in older females. In SAMP OVX estrogen loses its ability to modulate eNOS expression while significantly increases the expression of NOX1 and consequent production of superoxide (O_2_^−^). The fact that aged females (SAMP OVX) received immediate E2 replacement suggests that aging, rather than a long term estrogen withdrawn is responsible for the lack of estrogen-mediated effects observed. In agreement with our data, few studies have described loss of NO modulation by estrogen with aging [10]. However, this is the first study to describe a swap from antioxidant to pro-oxidant action by estrogen in association with vascular aging.

Most of estrogen action is mediated by two subtypes of estrogen receptors (ERα and ERβ). Although ERα has been described to be the receptor responsible for cardiovascular protection, insufficient and controversial information is available on the contribution of ERβ to the cardiovascular system[31]. Activation of ERα has been largely associated with increased eNOS transcription and NO production [19], [32], as well as its antioxidant mechanism [33], [34]. Studies on ER knockout (ERKO) mice have provided valuable clues into the tissue-specific roles of these receptors in cardiovascular physiology and pathophysiology. αERKO mouse have confirmed the importance of ERα in regulating endothelial NO synthesis. [35], [36]. On the other hand, mice lacking functional ERβ develop hypertension despite its normal NO production, suggesting that ERα is the main ER subtype responsible for NO production by estrogen[36]. Moreover, increased levels of ERβ have been associated with cardiovascular risk, including coronary calcification and atherosclerosis[31].

While much research focuses on ER-mediated NO production in the cardiovascular system, few studies have addressed the role of aging on ER balance and its correlation with estrogen-mediated effects. In this study we have hypothesized that a change on the balance of ERα and ERβ could account for the lack of vascular protection by estrogen. In our studies on ER protein expression and localization, when each ER subtype was individually correlated with the effects observed on NO and O_2_^−^ production a quite complex and confusing association can be made. However an important correlation between changes on the ratio ERβ/ERα and the regulation of NO and O_2_^−^ by aging was found. In our studies we have observed that aging itself did not affect ERβ/ERα in OVX animals, but markedly alters estrogen-mediated effects on ERs ratio. In young animals (SAMR OVX), although estrogen treatment produces a significant down-regulation of ER, it does not alter ERβ/ERα in both endothelium and vascular smooth muscle. The maintenance of ERβ/ERα is associated to beneficial effects by estrogen, i.e. up-regulation of NO production and decreased NAD(P)H oxidase activity and NOX1 expression. In contrast, aged females (SAMP OVX) exhibited a marked increase of ERβ/ERα following estrogen treatment, an effect that positively correlates to the reversal of beneficial action by estrogen on NO/O_2_^−^ balance. Corroborating our findings, a recent study have shown in prostate cancer that the action of estrogen on oxidative stress and the expression of antioxidant enzymes is dual and dependent on the ERα/ERβ ratio[37].

The mechanisms to explain how the imbalance of ER ratio reverses estrogen effects are largely unknown, but recent studies have interestingly revealed that ERβ exhibits an inhibitory action on ERα-dependent gene expression and may oppose the actions of ERα [38]. Moreover, a study by Bhavnani et al [39] has described different pattern of transcriptional activity by estrogens when using distinct ratios of ERα and ERβ in cells. Their results show that increasing ERβ concentration 2 or 10-fold inhibited the ERα activity progressively to levels that were 32 and 34% lower than those seen when ERα/ERβ ratio was 1∶1, indicating that the transcriptional activity of ERα is not only dependent on the concentration of estrogen, but also on concentrations of ERα/ERβ in a specific cell[39]. Although our data thus far is fairly descriptive, they provide an important hint on the relevance of ERα/ERβ equilibrium for estrogen-mediated effects on NO production and oxidative stress. More detailed and sophisticated protocols in biochemistry and molecular biology are needed to better characterize the specific role of different amounts of ERα and ERβ on the pharmacology of estrogens to regulate NO and O_2_^−^ production.

One process that has been increasingly proposed to link aging and the lack of cardiovascular protection is epigenetic modifications by vascular senescence [40]. Studies have suggested that aging-dependent increase in the methylation status of the promoter region of several genes that encode important regulators of cardiovascular function could be associated to the progression of cardiovascular disease, including atherosclerosis [40]. In this research, we further aimed to determine the occurrence and pathophysiological implications of aging-associated epigenetic alterations globally and in the promoter region of the gene that encodes ERα. Our data show a significant aging- associated increase on the percentage of global methylation in mice aorta. Surprisingly, we have also observed that estrogen also contribute to increase of DNA methylation in mice aorta, even though this effect cannot be associated to the changes observed herein. DNA methylation at 5′ flanking region of genes has been described as an important regulator of transcription, and a large body of evidence has demonstrated that increased DNA methylation is associated with decreased gene expression and even gene silencing [23]. Furthermore, an increased degree of ERα gene methylation appears to be associated to coronary atherosclerotic plaques and to vascular cell senescence [41]. Based on these principles, we have explored the hypothesis that aging-associated increase on the 5′flanking region of ERα could be associated to the decreased expression of this receptor and could account to the imbalance on ERβ/ERα. Using methylation-sensitive qPCR we observed that estrogen treatment is associated with increase on DNA methylation at ERα gene, an effect that could be involved into a negative feedback to control ER expression. However, these studies showed no aging-associated difference on DNA methylation on ERα gene. Both SAMR and SAMP from either OVX or estrogen-treated groups showed similar levels of DNA methylation at this site. Therefore, changes of DNA methylation status on the gene encoding ERα can explain estrogen-induced but not aging-associated decrease of ERα expression.

Taking together our studies have established that aging has a detrimental effect on estrogen-mediated regulation of NO and O_2_^−^ systems. An increase in the ratio between ERβ and ERα in older females is associated to the lack of the protective effects of estrogen on NO production and with a reversal in its antioxidant effect to a pro-oxidant profile. Although aged aortas display higher levels of DNA methylation globally, this epigenetic modification does not occur at the gene that encodes ERα and may be not associated to the swap on ER expression. Changes in arterial structure and function are the major vascular complication during aging and have been largely associated with increased risk of cardiovascular disease (such as atherosclerosis and hypertension) in the elderly. Our result improves our knowledge into the field of cardiovascular modulation by estrogens and establishes that aging significantly affects the direct estrogen-mediated mechanisms of vascular relaxation. Although the aging issue still needs to be better addressed in both experimental and clinical studies, our data demonstrate that estrogen has complex biologic effects and may influence the risk of cardiovascular events and other outcomes through multiple pathways. Therefore aging of a giving organism should always be taken into account when the pharmacological and physiological responses by estrogens are determined.
